# Financial toxicity among cancer patients, survivors and their families in the United Kingdom: a scoping review

**DOI:** 10.1093/pubmed/fdad143

**Published:** 2023-08-04

**Authors:** Tran T Ngan, Tran H Tien, Michael Donnelly, Ciaran O’Neill

**Affiliations:** Centre for Public Health*,* Queen’s University Belfast, Belfast BT12 6BA, UK; Department of Cancer Control and Population Health, National Cancer Center Graduate School of Cancer Science and Policy, Goyang 10408, Korea; Department of Pharmacy, University Medical Center Ho Chi Minh City, Ho Chi Minh City 700000, Vietnam; Centre for Public Health*,* Queen’s University Belfast, Belfast BT12 6BA, UK; Centre for Public Health*,* Queen’s University Belfast, Belfast BT12 6BA, UK

## Abstract

**Background:**

The aim of this scoping review was to identify key research gaps and priorities in order to advance policy and practice for people living with cancer in the UK.

**Methods:**

The review adhered to PRISMA guidelines for scoping review. We searched MEDLINE, EMBASE, Scopus, Web of Science and Google Scholar on 16 July 2022. There were no restrictions in terms of study design and publication time; gray literature was included. The key words, ‘financial’ or ‘economic’, were combined with each of the following words ‘hardship/stress/burden/distress/strain/toxicity/catastrophe/consequence/impact.’

**Results:**

29/629 studies/reports published during 1982–2022 were eligible to be included in the review. No study conducted a comprehensive inquiry and reported all aspects of financial toxicity (FT) or used a validated measure of FT. The most three commonly reported outcomes related to financial hardship were financial well-being (24/29), benefit/welfare (17/29) and mental health status (16/29).

**Conclusions:**

It is evident that FT is experienced by UK cancer patients/survivors and that the issue is under-researched. There is an urgent need for further research including rigorous studies which contribute to a comprehensive understanding about the nature and extent of FT, disparities in experience, the impacts of FT on outcomes and potential solutions to alleviate FT and related problems.

## Background

The term ‘financial toxicity’ (FT) is commonly used to refer to both the objective financial burden and the subjective financial distress experienced by cancer patients, survivors and their families as a result of cancer diagnosis and treatment.^1^^,^^2^ Objective financial burden stems from out-of-pocket payment (OOP) related to direct medical costs, direct non-medical costs (e.g. fuels for transportation, heating, special foods) and indirect non-medical costs (e.g. loss of income).^3^ Subjective financial distress, which is much more complex to assess, results from (i) the accumulation of OOP spending (direct from income or indirect through using savings or selling property); (ii) the concerns about the costs and how to deal with them and (iii) the challenge of changing behaviors and carrying out cost coping strategies (e.g. seeking financial assistance, reduce leisure activities).^3^^,^^4^

FT leads to a range of adverse financial, medical and social outcomes. Firstly and most obviously, the financial well-being of patients, survivors and their families may be negatively impacted by FT as they may lose savings and/or assets; have lower income and slower career development due to employment disruption during the cancer treatment; accumulate debts on credit cards; and fall behind on mortgage payments.^3^^,^^5^ Regarding health outcomes, lower health-related quality of life (HRQoL) has been mentioned by several studies on the topic.^4–7^ For example, in Italy where services are provided free at point of use by the state, a study with pooled data from 16 prospective multicentre trials reported 22.5% patients experienced FT that was significantly associated with an increased risk of death (Hazard Ratio (HR) 1.20, 95% confidence interval (CI) 1.05–1.37, *P* = 0.007).^7^ FT may also result in additional mental health distress and conditions such as depression and anxiety—the risk of developing these kinds of mental health problems is three times higher among cancer survivors who experience FT compared to cancer survivors without financial hardship.^8^

Media reports and voluntary sector bodies report the existence of FT among cancer patients in the UK and growing concerns regarding its effects in light of rapid increases in energy prices, rising inflation and interest rates. While the overwhelming and increasing cost of treatment, patient visits and prescriptions are covered by the government, all other direct non-medical and indirect costs still fall on the patients. Research has shown that individuals from the most socioeconomically disadvantaged groups such as lower income families, rural dwellers, minority groups, immigrants and young people are at greater risk of financial hardship.^5^^,^^8^ Despite the importance of the problem and growing interest, there is uncertainty about the nature and extent of FT studies in the UK.

This scoping review was conducted to review available published and gray literature about FT among cancer patients, survivors and their families in UK. The aims were to chart available empirical data about the topic of FT, identify the research gaps and key research priorities to advance policy & practice for people living with cancer in the UK.

## Methods

The conduct of this scoping review followed the methodological framework proposed by Arksey & O’Malley ^9^ and Levac *et al.*  ^10^ as well as the PRISMA guidance for the conduct and reporting of scoping reviews^11^ (See Appendix 1, Supplementary information for PRISMA-ScR checklist). There are five key stages to conducting a scoping review (plus optional stage 6).

### Stage 1: Identifying the research question

Our research question was, ‘What is known from existing literature about FT among cancer patients, survivors, and their families in the UK?’. The term ‘financial toxicity’ contains two sub-topics which are objective financial burden and subjective financial distress. According to the framework of FT proposed by Witte *et al.*^4^ subjective financial distress was further classified into three domains: (i) material conditions (e.g. the use of active and passive financial resources), (ii) psychological response (e.g. worries and concerns about their financial situation) and (iii) coping behaviors (to manage increased expenses). Preliminary search revealed that all aspects of FT are rarely researched in one study. Therefore, we decided to search for studies that reported data related to any aspect of these above sub-topics and domains in order to ensure the breadth of coverage.

### Stage 2: Identifying relevant studies

We performed the search in four bibliographic databases including MEDLINE, EMBASE, Scopus and Web of Science on 16 July 2022. Search inquiries did not apply a time limit or restrict any study type, but an English language-only restriction was applied. The results from initial searching indicated that the term ‘financial toxicity’ was not used commonly in the UK; therefore, we applied a wide range of alternative terms and a broad encompassing search strategy. The terms, ‘financial’ and ‘economic’ respectively were combined with hardship or stress or burden or distress or strain or toxicity or catastrophic or consequence or impact (See Table S1, Supplementary information for detailed database search strategies). To capture a wider range of study designs as well as gray literature, we searched Google Scholar and websites of relevant charity organizations including Macmillan Cancer Support, Cancer Now, Cancer Action and Young Lives versus Cancer. Additional potential papers were retrieved from the reference lists of included studies. Literature for which full text was not available (e.g. conference abstract) were excluded as information provided in an abstract is not enough to capture the full scope of an article and hinder the accuracy and quality of interpretation.

### Stage 3: Study selection

Selection criteria for studies were based on the PEO framework (PEO–Population|Exposure|Outcome) as follows: (i) Population: cancer patients (those who are under treatment), cancer survivors (those who finished initial treatment), and family members of cancer patients/survivors (whether or not they were providing informal care); (ii) Exposure: FT experienced by the population of interest; (iii) Outcome including financial well-being, HRQoL, mental health status and conditions (e.g. depression and anxiety), benefits/welfare, counseling service and any other support with a purpose that was to ease FT (See Table S2, Supplementary information for details of inclusion/exclusion criteria). Moreover, the setting was set to be United Kingdom; thus, only studies conducted among UK participants would be included.

All citations resulting from the searches were imported into web-based software platform Covidence. After removing duplicated citations, a selection process was conducted in two steps including (i) Title and abstract screening and (ii) Full-text review. Two reviewers (TTN and THT) independently conducted these two steps. In step 1, studies were moved to full-text review if at least one reviewer voted ‘included’. In step 2, when disagreement on study inclusion occurred, final inclusion was reached by consensus.

### Stage 4: Charting the data

A data charting form was developed and piloted by the research team using three randomly selected included studies and refined accordingly (See Appendix 2, Supplementary information). Two reviewers (TTN and THT) independently extracted data. Recorded information revolved around the PEO framework and the Witte *et al.* conceptualization of FT^4^ and included (i) General information (author(s) and their affiliation, year and type of publication, geographic coverage); (ii) Methods and participants (Objectives/research questions, study design, studied population); (iii) Exposure (FT) and outcomes (exposure definition or description, tools were used to measure it, outcomes of FT were studied) and (iv) Key findings.

### Stage 5: Collating, summarizing and reporting the results

We provide a descriptive numerical summary analysis of the extent, nature and distribution of the included studies to show the dominant areas of research. We then provide a qualitative thematic analysis in which findings from included reviews were organized and presented by different outcomes of FT. Finally, the implication of findings, the broader context and recommendations regarding future research are presented.

## Results

**Fig. 1:**
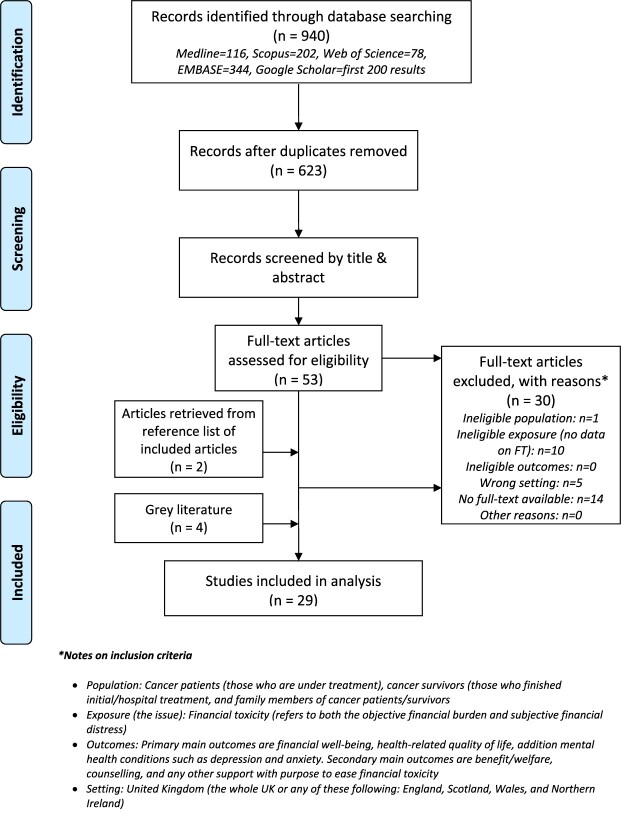
PRISMA flow diagram of literature search and selection.

We identified 740 citations from systematic searches on four databases and included the first 200 search results on Google Scholar. After removing duplicates, 623 citations were screened by title and abstract. 53 citations were moved to full-text review, of which, 23 were included^12–34^ (See Appendix 3, Supplementary information for full list of excluded reviews and justification for the exclusions). There were six additional studies (two peer-reviewed articles^35^^,^^36^ and four gray literature reports^37–40^ identified through manual searches of the reference lists of included citations and websites of relevant organizations. Therefore, a total of 29 studies were included in analysis^12–40^ (Fig. 1).

### Extent, nature and distribution of studies on FT

There was only one study^26^ that had as an explicit research objective to investigate FT. The focus of all other studies varied from the cost of cancer and its impact on family income and/or financial well-being to factors that influenced decisions about returning to work after treatment; from information/supportive needs of cancer patients to benefits/allowances that families were entitled to claim; from the general health and well-being of cancer survivors to the concerns/worries of cancer patients. No study reported all aspects of FT (objective financial burden and subjective financial distress).

Table 1 presents the numerical summary of the general information, methods and participants of 29 included studies.

**Table 1:** Overview of general information, methods and participants of 29 included studies

Category	Sub-category	*n*	%
Year of publication	Before 2001	1	3
	2001–2010	7	24
	2011–2022	21	72
Type of publication	Peer-reviewed article	24	83
	Gray literature	5	17
Geographic coverage	UK-Wide	10	34
	Great Britain	1	3
	England	14	48
	England & Scotland	1	3
	England & Wales	2	7
	Northern Ireland	1	3
Author(s)'s affiliations^a^	Academia	22	76
	Charity	5	17
	Hospital	6	21
	Others	1	3
Study design	Mixed methods	5	17
	Quantitative data (cross-sectional survey or secondary data analysis)	8	28
	Qualitative data	7	24
	Systematic review/review	7	24
	Others	2	7
Studied population^a^	Patients	15	52
	Survivors	7	24
	Carer/family members	7	24
Sample size (quantitative)	<200	5	38
	201–500	5	38
	500+	3	23
Sample size (qualitative)	<25	6	50
	25–60	5	42
	60+	1	8

a *Not mutually exclusive.*

### Design, participants, exposure and outcomes of studies on FT

In the only study that specifically investigated FT, patients were surveyed and classified as facing FT when they experienced greater financial burden at follow-up compared to their assessment at baseline.^26^ Financial burden was identified based on only one question (Q28) in the European Organization for Research and Treatment of Cancer (EORTC) core Quality of Life questionnaire (EORTC QLQ-C30) which asked patients to score financial difficulty relating to disease or treatment from 1 (not at all) to 4 (very much).^26^

No study used a validated instrument to assess FT such as ‘COST - The COmprehensive Score for financial Toxicity’.^41^ Most studies used bespoke questionnaire while some used generic instruments which cover a wide range of aspects related to cancer care such as Supportive Care Needs Survey-Short Form 34 (SCNS-SF34),^33^ EORTC QLQ-C30,^17^^,^^22^^,^^26^ and Social Difficulties Inventory.^17^^,^^22^

The most common outcomes related to financial hardship reported in included studies were financial well-being/situation (24/29 or 83%),^12^^,^^14^^,^^15^^,^^17–19^^,^^21–28^^,^^30^^,^^31^^,^^33–40^ benefit/welfare (17/29 or 59%),^12–14^^,^^16–19^^,^^21–23^^,^^25^^,^^27^^,^^28^^,^^30^^,^^31^^,^^36^^,^^40^ mental health (16/29 or 55%),^14–16^^,^^19^^,^^20^^,^^24^^,^^25^^,^^30–32^^,^^34–37^^,^^39^^,^^40^ employment after treatment (5/29 or 17%),^21^^,^^25^^,^^29^^,^^34^^,^^35^ and HRQoL (2/29 or 7%).^18^^,^^37^

### Key findings of studies on FT

Tables 2 and 3 provide summaries of included studies’ key findings. These findings are organized thematically into six following outcome-related themes.

**Table 2:** Summaries of included studies’ key findings (studies published before 2011)

Author Year	Objectives (aspects investigated)	Study design	Pop	Outcomes					Key findings: (a) Financial well-being, (b) Benefits/Welfare, (c) Mental health, (d) Employment, (e) HRQoL
				a	b	c	d	e	
Bodkin 1982^12^	Financial problems and hardship	Quan	C	x	x				a. Severe financial problems due to increased expenditure and loss of income b. Many families received financial help toward travel, special food, and heating from charitable sources. Most did not qualify for State benefits
Rozmovits 2003^32^	Information needs	Qual	P			x			c. Worries about loss of income
Chapple 2004^13^	Financial concerns, perceptions, and experiences with lung cancer	Qual	P		x				b. Unaware of financial benefits or lack of information on how to claim one. Stigma in claiming financial help.
Eiser 2006^36^	Costs of caring for a child with cancer; impact on parents’ income and the contribution of government benefits and charities	Quan	C	x	x	x			a. Changes in employment impacted negatively on finances of 42.7% families. Parents were forced to give up paid employment (34.7% mothers & 1.7% fathers), reduce working hours (28.7% mothers and 37.3% fathers) or changed employment (2% mothers and 1.7% fathers) b. Benefits were not received timely c. 68.3% families were worried about money. Lone parents had more financial concerns than parents who were married/cohabiting
Hanratty 2007^28^	Existence and consequences of financial stress and strain at the end of life for people dying with cancer	Sys rev		x	x				a. 16% to 80% claimed that they need more financial help. Differences among sociodemographic groups: 32% working class versus 16% middle class; 80% of the black carers versus 26% of the white carers b. 26% to 55% received attendance allowance
Kennedy 2007^29^	Factors that influence decisions to return to work and the experience of returning to work for cancer survivors	Qual	S				x		d. Primary reason (50%) for returning to work is financial pressure of being off work
Amir 2008^21^	How people have returned to the world of work	Qual	S	x	x		x		a. Built up significant debts on credit cards or fell behind with mortgage payments b. Dissatisfy with the financial protection for sick workers in the social welfare context of the UK c. Return to work earlier due to acute financial pressure
Moffatt 2010^14^	Impact of a welfare rights advice service specifically designed for people affected by cancer and their carers in County Durham, Northeast England (UK)	Qual	P + C	x	x	x			a. Most of the participants experienced financial strain following their cancer diagnosis. No financial impact was reported from households where a working partner earned a high income and/or the individual was well covered by private health insurance and/or mortgage protection. Financial impact was more severe for those of working age, especially those were self-employed b. Successful benefit claims was used to offset additional costs associated with cancer and lessen the impact of loss of earnings. Main barrier to access benefits was lack of knowledge about benefit entitlements c. Additional stress due to money worry. Quoted: ‘if you’ve got money worries it brings you down a little bit further’, ‘It’s a hard enough worry cancer itself, without having to worry about money as well’

*Study design—Mixed: Mixed methods, Qual: Qualitative research, Quan: Quantitative data, Rev: Narrative review, Sys rev: Systematic review.*

*Pop: Studies population—C: Carers/family members, P: Patients, S: Survivors.*

**Table 3:** Summaries of included studies’ key findings (studies published after 2010)

Author Year	Objectives (aspects investigated)	Study design	Pop	Outcomes					Key findings: (a) Financial well-being, (b) Benefits/Welfare, (c) Mental health, (d) Employment, (e) HRQoL
				a	b	c	d	e	
Brooks 2011^15^	Additional expenses related to cancer	Rev		x		x			a + c. Money worries increased for 68.3% families after diagnosis. Lone parents more likely to report money worries
Elliott 2011^24^	Self-reported health and well-being	Quan	S	x		x			a. 15–18% of cancer survivors were in debt but not worried about it a + c. 12–14% of cancer survivors with were in debt and worried about it
Young Lives vs Cancer 2011^39^	Additional costs facing families, how a cancer diagnosis disrupts their employment and ability to earn income, what financial support is available, and how families cope with these various impacts	Mixed	P + S + C	x		x			a + c. The number of parents who said that money was ‘often’ or ‘frequently’ a worry increased 8-fold after diagnosis, from 8% to 65%. 76% families said that childhood cancer had been a ‘big problem’ for their finances.
Amir 2012^35^	Effects of cancer’s related financial hardship/worries on family life (i.e. financial concerns of people affected by cancer)	Qual	P + C	x		x	x		a. Loss of income, especially for patients were in paid employment or self-employed at the time of diagnosis. Less or no impact on income of retired participants. Spend of savings, selling of possessions, altering usual activities and enjoyment of life to cope with loss of income c. Occurrence of negative emotions such as regret, disappointment, and self-reproach could lead to coexisting health problems and other difficulties. Family stress/strife, breakdown of relationships/families were other psychosocial facing patients and carers d. Return to work prematurely due to financial commitments. Concerns about job loss, employability, and lack of promotion.
Callanan 2012^23^	Benefits and allowances that families may be entitled to claim	Commentary		x	x				a. Increased financial burden due to loss of income and increased costs for special diet, new clothing, heating, travel, and car parking b. Financial support from state welfare benefit system were needed the most by people with limited or no income
Moffatt 2012^16^	Impact of welfare rights advice services on the quality of life and wellbeing of people with cancer	Mixed	P + C		x	x			b. Welfare benefits helped offset additional costs associated with cancer c. Receiving welfare benefits reduced levels of stress and anxiety related to financial difficulties.
Rogers 2012^17^	Financial burden of having head and neck cancer, and its relation with health-related quality of life (HRQoL)	Quan	P	x	x				a. 54% patients experienced at least one moderate or large financial burden. Greater financial difficulty due to loss of income. Younger people were more likely to experience financial difficulty b. 39% patients applied for benefits. Of those applied, 71% had received it. Patients in working age and men were more likely to apply for benefits
Rogers 2012^18^	Need for financial benefits, the advice patients were given about benefits and financial matters, and the financial burden of the disease	Quan	P	x	x			x	a. 57% reported that they were suffering financial hardship due to change in income b. 63% claimed that they need benefits. Unemployed (91%), part-time employed (71%), and those whose work was affected by cancer (75%) were more likely to need benefits. e. Decreased HRQoL (53%) as a result of the financial impact
Gardiner 2013^27^	Financial costs and the financial impact of caring for family members receiving palliative/end-of-life care	Sys rev		x	x				Included results from Hanratty *et al.* (2007)
Macmillan Cancer Support 2013^38^	Financial impact cancer is having on people across the UK	Mixed	P + S	x					a. 83% people are financially affected. The household was on average £570 a month worse off. Key factors that negatively influenced the severity of financial hardship were younger age (<60 years old), undergone chemotherapy and/or surgery, self-employed or part-time employed, and low income
McGarry 2013^30^	Unmet supportive needs of people with breast cancer attending a London NHS Foundation Trust Hospital	Mixed	P	x	x	x			a. 17% of participants had concerns about finance (e.g. difficulties with rent and bills) due to inability to work or reducing of working hours b. Support from the system was insufficient to meet patient’s need and they had to depend on family for additional support c. Financial concerns added to overall stress during treatment
Azzani 2015^22^	Prevalence of perceived financial hardship and associated factors	Sys rev		x	x				Included results from Rogers *et al.* (2012) ‘impact’
Moffatt 2015^19^	Connections between cancer and employment; specifically, decisions, choice and constraints around returning to work or remaining outside the labor force	Qual	P	x	x	x			a. Affect household finances due to significant drop in income. Coping strategies are using savings, borrowing cash, cut on household expenditure, and selling property b. Claiming welfare, even for a cancer-related illness, is stigmatizing. c. It was stressful due to concern over the impact of cancer on financial situation, future employment prospects, and families’ life
Pelletier 2015^31^	Family financial burden in childhood cancer	Sys rev		x	x	x			Included results from Eiser *et al.* (2006)
Young Lives vs Cancer 2016^40^	Additional costs facing young cancer patients and their families; how a cancer diagnosis is disrupting the employment and income; emotional impact of the financial burden of cancer	Mixed	P + C	x	x	x			a. Parents spent £600 extra per month during active treatment of their children. For young patients, it was £360/month. Great financial pressure: 61% had built up debt and 17% borrowed over £5000 b. Forms to apply for Disability Living Allowance and Personal Independence Payment was long and stressful to complete, and patients often required help to fill out (84%) c. 76% of parents and 54% of young people reported additional stress and anxiety while managing their finances during treatment
Macmillan Cancer Support 2017^37^	Financial impact of cancer		P + S	x		x		x	a. 39% of people with cancer have used savings, sold assets or borrowed to cover the costs or the loss of income caused by their diagnosis. 30% carers reported that their income or household finances were affected by caring c. 53% reported feeling more anxious or stressed. 37% said it had made them feel more isolated or alone e. Negatively affected quality of life (61%)
Watson 2019^33^	Care experiences and supportive care needs	Quan	P	x					a. Negatively impacted on day-to-day financial situation (51%)
Flaum 2020^26^	FT and financial burden	Quan	P	x					a. Prevalence of FT among surveyed cancer patient was reported at 20%
Zhu 2020^34^	Cancer survivors’ experiences with FT	Sys rev		x		x	x		Included results from Amir *et al.* (2012)
Lu 2021^20^	Association between levels of financial stress and cancer-related fatigue (CRF)	Quan	S			x			c. 11% survivors reported both pre- and post-diagnosis financial stress (cumulative stress). Survivors with cumulative financial stress exposure were significantly more likely to have CRF (Odds ratio (OR) = 4.58, 95% CI 3.30–6.35, *P* < 0.001), compared with those without financial stress.
Fitch 2022^25^	Cancer-related FT or burden	Sys rev		x	x	x	x		Included results from Moffatt *et al.* (2010), Moffatt *et al.* (2012) and Amir *et al.* (2012)

*Study design—Mixed: Mixed methods, Qual: Qualitative research, Quan: Quantitative data, Rev: Narrative review, Sys rev: Systematic review.*

*Pop: Studies population—C: Carers/family members, P: Patients, S: Survivors.*

#### Impact on financial well-being

Most studies (24/29) reported that the patients, survivors and/or carers faced severe financial problems following their cancer diagnosis.^12^^,^^14^^,^^15^^,^^17–19^^,^^21–28^^,^^30^^,^^31^^,^^33–40^ These problems manifested in varied forms such as being in debt,^21^^,^^24^ difficulties paying rent/bills/mortgage,^21^^,^^30^^,^^33^ needing financial help,^19^^,^^28^^,^^37^ spending savings,^19^^,^^35^^,^^37^ selling possessions,^19^^,^^35^^,^^37^ altering usual activities and enjoyment of life to cope.^19^^,^^35^ The two main reasons leading to such situations were loss of income (e.g. due to needing to stop working or reduce working hours) and additional direct non-medical costs (e.g. special diet, heating, travel and car parking).

Only 2/24 studies quantified the loss of income and extra expenditure. Macmillan’s study reported that households, on average, were £570/month worse off following a diagnosis of cancer.^38^ Young Lives versus Cancer studies reported that parents spent extra £600/month during active treatment of their children while young cancer patients spent £360 extra per month.^40^

Few studies (5/24) reported the disparities among sociodemographic groups that financial impact was more severe for those of working age, especially self-employed or part-time employed^14^^,^^17^^,^^38^; lone parents^15^; and among those who belonged to minority ethnic groups.^28^

#### Impact of benefit/welfare system

Nearly two-thirds of studies (17/29) detailed the experiences regarding the benefit/welfare system.^12–14^^,^^16–19^^,^^21–23^^,^^25^^,^^27^^,^^28^^,^  ^30^^,^^31^^,^^36^^,^^40^ Financial burden resulted in cancer patients applying for benefits such as attendance allowance, disability attendance allowance and/or personal independence payment^17^^,^^28^^,^^40^ even though there was stigma associated with applying.^13^^,^^19^ Studies also reported patients’ dissatisfaction toward the benefit system—they complained that the application process was complicated and lengthy,^21^^,^^36^^,^^40^ the benefits that they received were inadequate,^30^ and there was an overall lack of information about benefit entitlements.^13^^,^^14^ All these issues added to the stress felt by patients.

#### Impact on mental health

More than half of studies (16/29) reported how financial burden and struggles with obtaining benefits affected mental health of patients, survivors and/or carers.^14–16^^,^^19^^,^^20^^,^^24^^,^^25^^,^^30–32^^,^^34–37^^,^^39^^,^^40^ The most common aspect reported was ‘worry about money’ which led to additional stress.^14^^,^^19^^,^^30^^,^^37^^,^^40^ Lone parents were more likely to report money worries.^15^^,^^36^ Occurrence of negative emotions such as regret, disappointment and self-reproach about their financial situation was viewed as leading to coexisting health problems and other difficulties.^35^ Family stress/strife, breakdown of relationships/families were other significant psychosocial challenges facing patients and carers.^35^

#### Impact on employment during and after treatment

Few studies (5/29) reported this impact in a way that patients had to return to work prematurely due to financial pressure as a result of being off work.^21^^,^^25^^,^^29^^,^^34^^,^^35^

#### Impact on HRQoL

This was reported by only 2/29 studies.^18^^,^^37^ Macmillan’s study reported that the HRQoL of 61% of patients was negatively affected though the validity of the method to measure HRQoL was unclear.^37^ Rogers *et al.* reported that 53% of patients who suffered financially had decreased HRQoL as measured by the University of Washington Quality of Life questionnaire.^18^

## Discussion

### Main findings of this study

The significant increase in the number of publications on the subject in recent years reflects a growth of interest in the issue of FT within the field of cancer research. However, no study in the UK has investigated FT as this term is commonly understood. Objective financial burden and subjective financial distress were not clearly delineated in any study. As a result, we needed to adjust and broaden the inclusion criteria of the scoping review in order to include studies that reported any aspect of FT.

Publications came mostly from authors in academia though there were contributions from charity organizations and/or hospital Trusts. Indeed, charity organizations have published their own reports about the financial impact of cancer. These reports appeared to indicate a stronger presence of a wide variety of aspects related to financial impact than the peer-reviewed articles that tended to focus on only one aspect. Collaboration between the voluntary sector and academics may help bring additional rigor to such studies and give a greater degree of credibility to these types of reports.

A key limitation is that all quantitative studies on FT used retrospective data. There has not been a study in the UK that has used prospective data to investigate the issue of FT among cancer patients. A prospective cohort study following patients from the point of diagnosis to finish initial treatment would provide invaluable insights to the causes and effects of FT on cancer patients. Such studies may be less likely to be subject to recall bias as well as providing the opportunity to study the relationship between FT and cancer as treatment progresses and/or economic conditions change.

The most studied population was cancer patients. Studies have paid some attention to survivors and carers/family members though they tended to be studied separately. Future research should assess the FT situation from the perspective of all key parties (i.e. patients, survivors, carers/family members) as well as the views of other stakeholders such as the community and voluntary sector. The involvement of one or more charity organizations in the recruitment process and the associated larger sample sizes in these studies points to the importance of adopting a collaborative approach in future research.

The validated questionnaire to investigate FT, COST, has not been used in any UK studies. Authors often used bespoke questionnaire or generic instrument which was not specialized for the issue of FT. It is recommended that future research should use COST to improve research rigor and facilitate the comparison of results with similar research around the world.

The majority of studies focused on describing objective financial burden and its material impact on the financial well-being of cancer patients, survivors, and/or carers/family members. Subjective financial distress, especially its psychosocial effects, is under-researched. There is a need to give research attention to investigating disparities between different sociodemographic groups. The review found that studies are sparse regarding the causes of financial stresses and strain. While some FT-related outcomes were investigated, there is a need to assess FT using psychometrically validated instruments. These critical gaps for future research need to be addressed in order to plan person-centered service responses for patients who encounter FT.

### What is already known on this topic

FT exists and has now become a serious issue in high income countries with publicly funded health system and universal coverage.^8^^,^^42^^,^^43^ Studies from Canada, Italy, Germany and Japan have reported significant prevalence of FT among cancer patients/survivors as well as its impact on health outcomes.^7^^,^^43–45^

### What this study adds

There exists a paucity of research on FT among cancer patients, survivors and their families in the UK. Current evidence is ad hoc and at times anecdotal with studies using different definitions, methods and studying often only small parts of the overall issues. Nevertheless, that FT exists in the UK is evident.

The scoping review also identified key research gaps and suggested priorities for future research. As such, a comprehensive study designed to provide a better understanding about the nature and extent of the problem, disparities in experience (among different sociodemographic groups and types of cancer), the impacts of FT on outcomes, and potential solutions to alleviate FT and related problems is urgently needed.

### Limitations of this study

Our comprehensive and systematic approach to identification, selection, data charting and analysis followed the rigorous methodological framework set out by Arksey & O’Malley and Levac *et al.*  ^9^^,^^10^ and PRISMA guidance for the conduct and reporting of scoping reviews.^11^ However, due to time constraint, we could not conduct and include (optional) stage 6 of the methodological framework (i.e. consultation exercise) in this paper.

## Acknowledgments

The authors would like to thank Mr Richard Fallis who is the subject librarian of the School of Medicine, Dentistry & Biomedical Sciences at Queen’s University Belfast for his advice on the development of the search strategy.

## Supplementary information

This contains two tables and three appendices as following, Table S1—Detailed search strategies for all databases/search engine; Table S2—Detailed inclusion and exclusion criteria; Appendix 1—PRISMA extension for scoping reviews (PRISMA-ScR) checklist; Appendix 2: Data charting template (used in Covidence); Appendix 3—List of excluded reviews and justification for the exclusions.

## Funding information

This work was supported by the Wellcome Trust Early Career Award [grant number 226921/Z/23/Z]. The corresponding author had full access to all the data in the study and had final responsibility for the decision to submit for publication.

## Conflicts of interest

The authors declare that they have no competing interests.

## Data availability

Search strategies needed to replicate the study are included in Table S1, Supplementary information.

## Authors’ contributions

TTN, CON, and MD conceived and designed the scoping review. TTN and THT participated in the study selection and data extraction. All authors contributed to the interpretation of the findings. TTN wrote the first draft and prepared the manuscript. MD and CON provided supervisory support and reviewed this paper. All authors contributed to the revision of the manuscript and approved the final version of the review.

## Supplementary Material

Supplementary_information_JPH_fdad143

## Notes

**Tran T. Ngan,** PhD

**Tran H. Tien,** MSc

**Michael Donnelly,** PhD

**Ciaran O’Neill,** PhD
